# Emergency general surgery: impact of distance and rurality on mortality

**DOI:** 10.1093/bjsopen/zrac032

**Published:** 2022-04-25

**Authors:** Jared M. Wohlgemut, George Ramsay, Mohamed Bekheit, Neil W. Scott, Angus J. M. Watson, Jan O. Jansen

**Affiliations:** Centre for Trauma Sciences, Blizard Institute, Queen Mary University of London, London, UK; General Surgical Department, Aberdeen Royal Infirmary, Aberdeen, UK; Health Services Research Unit, University of Aberdeen, Aberdeen, UK; General Surgical Department, Aberdeen Royal Infirmary, Aberdeen, UK; Department of Surgery, Elkabbary Hospital, Alexandria, Egypt; Medical Statistics Team, University of Aberdeen, Aberdeen, UK; Department of Surgery, Raigmore Hospital, Inverness, UK; Division of Trauma and Acute Care Surgery, Department of Surgery, University of Alabama at Birmingham, Birmingham, Alabama, USA

## Abstract

**Background:**

There is debate about whether the distance from hospital, or rurality, impacts outcomes in patients admitted under emergency general surgery (EGS). The aim of this study was to determine whether distance from hospital, or rurality, affects the mortality of emergency surgical patients admitted in Scotland.

**Methods:**

This was a retrospective population-level cohort study, including all EGS patients in Scotland aged 16 years or older admitted between 1998 and 2018. A multiple logistic regression model was created with inpatient mortality as the dependent variable, and distance from hospital (in quartiles) as the independent variable of interest, adjusting for age, sex, co-morbidity, deprivation, admission origin, diagnosis category, operative category, and year of admission. A second multiple logistic regression model was created with a six-fold Scottish Urban Rural Classification (SURC) as the independent variable of interest. Subgroup analyses evaluated patients who required operations, emergency laparotomy, and inter-hospital transfer.

**Results:**

Data included 1 572 196 EGS admissions. Those living in the farthest distance quartile from hospital had lower odds of mortality than those in the closest quartile (OR 0.829, 95 per cent c.i. 0.798 to 0.861). Patients from the most rural areas (SURC 6) had higher odds of survival than those from the most urban (SURC 1) areas (OR 0.800, 95 per cent c.i. 0.755 to 0.848). Subgroup analysis showed that these effects were not observed for patients who required emergency laparotomy or transfer.

**Conclusion:**

EGS patients who live some distance from a hospital, or in rural areas, have lower odds of mortality, after adjusting for multiple covariates. Rural and distant patients undergoing emergency laparotomy have no survival advantage, and transferred patients have higher mortality.

## Teaser

The aim of this study was to determine whether distance from hospital, or rurality, affects the mortality of Scottish emergency general surgery (EGS) patients. The study found that EGS patients who live some distance from a hospital, or in rural areas, have lower odds of mortality, after adjusting for multiple covariates. Rural and distant patients undergoing emergency laparotomy have no survival advantage, and transferred patients have higher mortality.

## Introduction

The impact of distance from hospital, and rurality, on mortality in emergency general surgery (EGS) patients remains unclear, with previous studies demonstrating a range of effects, from beneficial, to harmful^1–9^. Travel time to hospital was not a primary determinant of mortality in laparotomy audits in Britain or rural Australia^1,4^. Further studies demonstrated that it is safe to provide EGS laparotomies in non-urban centres in the USA and Australia^3,5,6^. In Scotland, one study showed distance was not related to mortality after ruptured abdominal aortic aneurysm^8^. A later Scottish study demonstrated decreased mortality with greater distance from the hospital but admitted the possibility of survival bias in their methodology^9^. In summary, the evidence is at best inconclusive, and at worst contradictory.

Many studies define EGS patients as those who have undergone an emergency operation. However, as less than 25 per cent of patients admitted under surgical services as an emergency undergo an operation, it is helpful to define EGS patients as all non-scheduled admissions under the care of a general surgeon^10^. It is also important to recognize that irrespective of whether patients live in an urban or rural setting, they may live very close to, or far away from an admitting EGS hospital. Therefore, it is useful to investigate both rurality and distance from hospital. It is not known whether patients who require an EGS admission are more likely to survive based on the distance from hospital or rurality. This question has profound implications for service delivery.

The aim of this study was to determine whether distance from hospital, or rurality, affects mortality of EGS patients in Scotland. Scotland has large remote and rural areas, particularly in the North and West of the country, and many islands. The hypothesis was that mortality increases in EGS patients as distance between home and hospital increases, and as rurality increases.

## Methods

### Design

This was a retrospective population-level cohort study.

### Data source

Administrative data from the Information Services Division of the Government of Scotland were routinely collected. This national database included population-level data of EGS patients during the study interval.

### Population

An EGS patient was defined as a patient aged 16 years and older, non-electively admitted to a Scottish hospital, under the care of a consultant (attending) general surgeon for the full calendar years of 1998–2018 inclusive. Patients were followed up for 6 months.

### Setting

Scotland has a national healthcare system where patients are treated at no direct cost to the patient. EGS care is provided by general surgeons working at teaching hospitals, large district general hospitals, and small district general hospitals^11^.

### Data extracted

Data extracted included age at admission, sex, Charlson co-morbidity index (CCI, 10-year look-back), Scottish index of multiple deprivation (SIMD), admission origin (from home (domicile), transferred from another hospital, or other—including nursing homes, prisons, or no fixed abode), diagnosis (coded by use of the ICD-10)^12^, operations (coded by use of the OPCS-4)^13^, distance from hospital (calculated as the distance of a straight line between patient address and hospital address), year of admission, and date of death. SIMD is a measure of socioeconomic deprivation, comprehensively ranking all small geographical areas in Scotland (based on income, employment, education, health, access to services, crime, and housing), and then further classifying them in quintiles, with 1 as the most deprived and 5 as the least deprived^14^.

Age was categorized by 15-year increments (16–30, 31–45, 46–60, 61–75, and more than 75 years); co-morbidity into none (CCI of 0), mild (CCI of 1–2), moderate (CCI of 3–4), and severe (CCI more than 4); diagnosis into high- or low-risk diagnosis (based on the classification by Symons, *et al*.; *Table S1*)^15^; treatment into non-operative, operative laparotomy (OPCS-4 Y50.2 and T30), operative laparoscopy (OPCS-4 Y75), operative other gastrointestinal (not OPCS-4 G, H, or J), operative skin/soft tissue (OPSC-4 S and T, except T30), operative other non-gastrointestinal (OPCS-4 A–F; K–R, and V–X); and distance from hospital into quartiles (0–2.9 km, 2.9–6.4 km, 6.4–15.2 km, and more than 15.2 km). These categorizations and their justifications are also based on previously published work^10,16–21^.

### Outcome

Mortality data were obtained as inpatient mortality (death before discharge from hospital), and 1-year mortality (death within 1 year of discharge from hospital). Inpatient mortality may be more representative of hospital performance, whereas 1-year mortality may better reflect healthcare system functioning. Outcome data were linked via patients’ unique community health index number^22^.

### Analysis

**Fig. 1:**
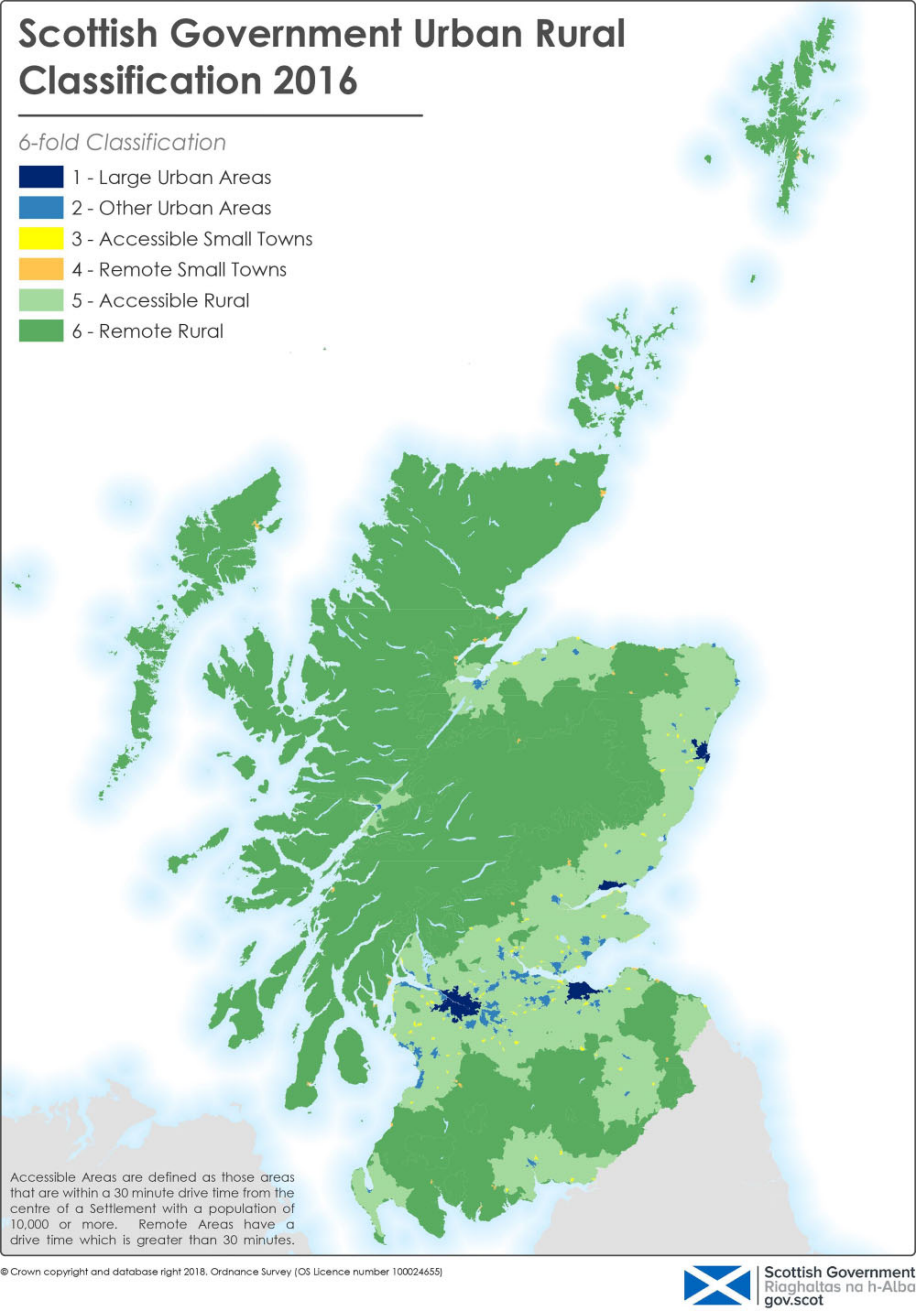
**Scottish Government six-fold urban rural classification 2016**. Reproduced with permission^23^

The data were analysed with two logistic regression models. The first model explored the effect of distance to hospital, adjusting for variables chosen *a priori* that were previously shown to significantly affect the outcome of interest (mortality)^19,20^. The model defined inpatient mortality as the dependent variable, and distance from hospital as the independent variable of interest, adjusting for age, sex, co-morbidity (CCI), deprivation (SIMD), admission origin, diagnosis category, operative category, and year of admission. An identical model was created with 1-year mortality as the dependent variable. The second model explored the impact of rurality, with the six-fold SURC as the independent variable of interest. An identical model with 1-year mortality as the dependent variable was also analysed. The SURC incorporates several factors, and ‘provides a consistent way of defining urban and rural areas across Scotland’^23^. Several versions are available, with varying numbers of categories, ranging from two to eight. The latest (eight-fold) version was published in 2016. Previous research has shown that within the eight-fold classification, category 5 residents have very short travel times to hospitals^24^. Therefore, the six-fold classification was chosen as the most detailed SURC available without inconsistency regarding travel times (*Table S2* and *Fig. 1*). Sensitivity analyses were repeated with the three-fold SURC, and two-fold SURC. Analyses were conducted with SPSS^®^ version 27 (IBM, Armonk, New York, USA).

### Subgroup analyses

Analyses were repeated for several predefined subgroups, including patients who underwent operative treatment (*Tables S3* and *S4*), patients who required emergency laparotomy (*Tables S5* and *S6*), and patients who were transferred to a higher level of care (*Tables S7* and *S8*).

### Study conduct

This study was approved via the Public Benefit and Privacy Panel for Health and Social Care (PBPP 1819-0340) and did not require further research ethics approval. The STROBE guidelines were used to inform manuscript preparation (*Table S9*)^25^.

## Results

There were 1 631 198 patients admitted to emergency general surgical services during the study interval. We excluded 48 351 who were aged under 16 years, 14 with missing sex data, and 10 637 whose place of residence could not be assigned to an SURC category, leaving a total of 1 572 196 admissions. *Table 1* outlines the baseline characteristics of the population cohort by distance from hospital. We found that more young people lived closer to their admitting hospital than older patients. Sex proportions were similar across distance quartiles. A higher proportion of patients with moderate and severe co-morbidity lived further from their admitting hospital. Patients from deprived geographical areas (SIMD 1) were more likely to live close to their admitting hospital, whereas more of those in medium levels of deprivation (SIMD 3 and 4) lived further away. More patients were transferred who lived further away. A higher proportion of patients with high-risk diagnoses lived distant from their admitting hospital. Non-operative treatment was similar by distance quartile, but more laparotomies were performed for patients living further away.

**Table 1:** Demographics and inpatient mortality by distance from hospital (quartiles), for Scottish emergency general surgery admissions 1998–2018

Demographic	Category	Distance from hospital			
		0–2.9 km *n* (%)	2.9–6.4 km *n* (%)	6.4–15.2 km *n* (%)	>15.2 km *n* (%)
**Age category**	16–30 years	78 747 (27)	75 671 (26)	71 191 (24)	66 378 (23)
	31–45 years	84 688 (26)	85 777 (26)	81 611 (25)	73 949 (23)
	46–60 years	81 368 (24)	85 868 (25)	87 589 (26)	85 598 (25)
	61–75 years	79 950 (23)	81 553 (24)	86 469 (25)	92 486 (27)
	More than 75 years	68 442 (25)	64 052 (23)	66 210 (24)	74 599 (27)
**Sex**	Male	188 744 (25)	183 467 (25)	181 240 (24)	189 631 (26)
	Female	204 451 (25)	209 454 (25)	211 830 (26)	203 379 (25)
**CCI 10 year**	0; no co-morbidity	227 076 (25)	227 321 (25)	227 880 (25)	214 372 (24)
	1–2; mild co-morbidity	106 957 (25)	105 305 (25)	104 306 (25)	108 675 (26)
	3–4; moderate co-morbidity	31 148 (24)	31 365 (24)	31 317 (24)	34 972 (27)
	>4; severe co-morbidity	28 014 (23)	28 930 (24)	29 567 (24)	34 991 (29)
**SIMD quintile**	1	143 429 (31)	152 139 (33)	106 983 (23)	56 676 (12)
	2	96 692 (27)	88 612 (24)	90 723 (25)	87 063 (24)
	3	61 793 (20)	57429 (19)	72 684 (24)	114 227 (37)
	4	45 900 (18)	44 953 (18)	68 690 (27)	91 290 (36)
	5	45 381 (24)	49 788 (26)	53 990 (28)	43 754 (23)
**Origin**	Domicile	377 461 (25)	380 863 (25)	381 115 (25)	364 932 (24)
	Other	12 028 (25)	8818 (18)	8155 (17)	19 388 (40)
	Transfer	1582 (13)	1813 (15)	2413 (20)	6061 (51)
**High-risk diagnosis**	No	368 095 (25)	365 576 (25)	364 277 (25)	359 141 (25)
	Yes	25 100 (22)	27 345 (24)	28 793 (25)	33 869 (29)
**Treatment categories**	Non-operative	227 065 (25)	232 514 (26)	236 049 (26)	209 563 (23)
	Operative GI other	61 944 (24)	63 910 (24)	65 382 (25)	71 451 (27)
	Operative other non-GI	68 040 (27)	58 806 (23)	54 259 (22)	71 127 (28)
	Operative skin/soft tissue	*	23 121†	21 858†	*
	Operative laparoscopy	*	9503†	10 555†	*
	Operative laparotomy	4720 (23)	5067 (24)	4967 (24)	6010 (29)
**Inpatient mortality**	Alive	386 530 (25)	386 471 (25)	386 758 (25)	386 957 (25)
	Dead	6665 (26)	6450 (25)	6312 (25)	6053 (24)

* Cannot calculate percentage. CCI, Charlson co-morbidity index; SIMD, Scottish index of multiple deprivation (1, most deprived; 5, least deprived); GI, gastrointestinal; SURC, Scottish Urban Rural Classification.

† Number is less than or equal to 5, or in the same row of a number that is less than or equal to 5, which may be identified.

*Table 2* outlines the baseline characteristics by six-fold SURC. There were many more patients who lived in urban geographical areas (SURC 1 and 2) than rural locations (SURC 3–6) (*Table 2*). There were similar proportions of age group, sex, co-morbidity, origin, high-risk diagnosis, and treatment categories by SURC category; however, patients living in high levels of deprivation (SIMD 1 and 2) tended to live in urban areas (SURC 1 and 2), whereas a higher proportion of people living in medium deprivation regions (SIMD 3 and 4) lived in more-rural areas (*Table 2*).

**Table 2:** Demographics and inpatient mortality by six-fold Scottish urban/rural classification, for Scottish emergency general surgery admissions 1998–2018

Demographics	Category	Six-fold SURC					
		1 *n* (%)	2 *n* (%)	3 *n* (%)	4 *n* (%)	5 *n* (%)	6 *n* (%)
**Age category**	16–30 years	110 771*	110 743*	21 287*	†	†	†
	31–45 years	119 714*	126 130*	23 947*	†	†	†
	46–60 years	116 765 (34)	128 426 (38)	26 161 (8)	13 830 (4)	33 393 (10)	21 848 (6)
	61–75 years	108 135 (32)	127 769 (38)	27 736 (8)	16 005 (5)	34 441 (10)	26 372 (8)
	More than 75 years	87 441 (32)	98 470 (36)	22 148 (8)	15 712 (6)	25 654 (9)	23 878 (9)
**Sex**	Male	254 533 (35)	276 886 (37)	56 414 (8)	34 166 (5)	69 718 (9)	51 365 (7)
	Female	288 293 (35)	314 652 (38)	64 865 (8)	35 676 (4)	74 279 (9)	51 349 (6)
**CCI 10 year**	0; no co-morbidity	315 764 (35)	335 646 (37)	68 475 (8)	37 902 (4)	83 145 (9)	55 717 (6)
	1–2; mild co-morbidity	144 294 (34)	161 234 (38)	32 831 (8)	19 933 (5)	38 087 (9)	28 864 (7)
	3–4; moderate co-morbidity	43 379 (34)	48 549 (38)	10 099 (8)	6382 (5)	11 146 (9)	9247 (7)
	>4; severe co-morbidity	39 389 (32)	46 109 (38)	9874 (8)	5625 (5)	11 619 (10)	8886 (7)
**SIMD quintile**	1	233 368 (51)	184 190 (40)	20 342 (4)	9054 (2)	8912 (2)	3361 (1)
	2	104 179 (29)	171 271 (47)	26 948 (7)	21 299 (6)	23 638 (7)	15 755 (4)
	3	63 546 (21)	101 726 (33)	29 877 (10)	20 131 (7)	43 209 (14)	47 644 (16)
	4	56 917 (23)	67 256 (27)	21 964 (9)	15 503 (6)	55 593 (22)	33 600 (13)
	5	84 816 (44)	67 095 (35)	22 148 (11)	3855 (2)	12 645 (7)	2354 (1)
**Origin**	Domicile	527 351 (35)	565 524 (38)	116 088 (8)	64 096 (4)	136 999 (9)	94 313 (6)
	Other	12 089 (25)	17 765 (37)	3505 (7)	4321 (9)	4706 (10)	6003 (12)
	Transfer	1683 (14)	5718 (48)	966 (8)	755 (6)	1231 (10)	1516 (13)
**High-risk diagnosis**	No	506 554 (35)	547 354 (38)	111 399 (8)	65 028 (4)	131 693 (9)	95 061 (7)
	Yes	36 272 (32)	44 184 (38)	9880 (9)	4814 (4)	12 304 (11)	7653 (7)
**Treatment categories**	Non-operative	323 039 (36)	345 096 (38)	68 643 (8)	37 675 (4)	78 608 (9)	52 130 (6)
	Operative GI other	86 602 (33)	102 094 (39)	21 837 (8)	10 083 (4)	25 954 (10)	16 117 (6)
	Operative other non-GI	80 586 (32)	87 689 (35)	18 686 (7)	16 056 (6)	24 125 (10)	25 090 (10)
	Operative skin/soft tissue	32 492*	34 626*	†	†	†	†
	Operative laparoscopy	13 241*	14 819*	†	†	†	†
	Operative laparotomy	6866 (33)	7214 (35)	1698 (8)	998 (5)	2398 (12)	1590 (8)
**Inpatient mortality**	Alive	534 265 (35)	581 704 (38)	119 257 (8)	68 719 (4)	141 785 (9)	100 986 (7)
	Dead	8561 (34)	9834 (39)	2022 (8)	1123 (4)	2212 (9)	1728 (7)

* Cannot calculate percentage.

† Number is less than or equal to 5, or in the same row of a number that is less than or equal to 5, which may be identified. SURC, Scottish Urban Rural Classification; CCI, Charlson co-morbidity index; SIMD, Scottish index of multiple deprivation (1,  most deprived; 5,  least deprived); GI, gastrointestinal.

*Table S10* displays rates of inpatient mortality based on distance from hospital (in quartiles), by age category, sex, co-morbidity, deprivation, origin, diagnosis categories, treatment categories, and six-fold SURC. A higher proportion of patients died who lived closer to the admitting hospital, who were older, female, highly co-morbid, admitted from non-domicile environments, with high-risk diagnosis, and who had a laparotomy. *Table S11* demonstrates inpatient mortality for each six-fold SURC category.

### Distance from hospital and mortality

Those admissions of patients who lived in the furthest quartile from hospital had lower odds of mortality than those in the closest quartile (OR 0.829, 95 per cent c.i. 0.798 to 0.861), although there was no statistically significant difference between the first and second, or first and third quartiles (*Table 3*). With 1-year mortality as the dependent variable, those in the second and fourth quartiles of distance from hospital had higher odds of survival than those closest to hospital (OR 0.982, 95 per cent c.i. 0.966 to 0.998; OR 0.920, 95 per cent c.i. 0.905 to 0.935 respectively) (*Table 3*).

**Table 3:** Multiple logistic regression: inpatient and 1-year mortality as dependent variable, with distance from hospital (quartiles) as covariate of interest, for all admissions

		Inpatient mortality as dependent			1-year mortality as dependent		
		OR	95% c.i.	*P*	OR	95% c.i.	*P*
**Distance from hospital**	0–2.9 km (reference)	1			1		
	2.9–6.4 km	1.012	0.976 to 1.049	0.522	0.982	(0.966 to 0.998)	0.026
	6.4–15.2 km	0.991	0.955 to 1.028	0.625	1.007	(0.991 to 1.024)	0.383
	>15.2 km	0.829	0.798 to 0.861	<0.001	0.92	(0.905 to 0.935)	<0.001
**Age category**	16–30 years (reference)	1			1		
	31–45 years	2.88	2.296 to 3.611	<0.001	3.221	(3.042 to 3.41)	<0.001
	46–60 years	9.009	7.296 to 11.124	<0.001	7.488	(7.094 to 7.904)	<0.001
	61–75 years	21.442	17.409 to 26.41	<0.001	15.183	(14.393 to 16.016)	<0.001
	More than 75 years	49.904	40.537 to 61.435	<0.001	32.435	(30.751 to 34.212)	<0.001
**Sex**	Female (male is reference)	1.123	1.094 to 1.153	<0.001	0.892	(0.882 to 0.902)	<0.001
**CCI 10 year**	0; no co-morbidity (reference)	1			1		
	1–2; mild co-morbidity	3.497	3.335 to 3.668	<0.001	3.367	(3.313 to 3.423)	<0.001
	3–4; moderate co-morbidity	5.838	5.545 to 6.147	<0.001	6.191	(6.074 to 6.31)	<0.001
	>4; severe co-morbidity	14.115	13.455 to 14.807	<0.001	22.905	(22.487 to 23.331)	<0.001
**SIMD quintile**	5 (reference)	1			1		
	4	1.091	1.037 to 1.148	0.001	1.02	(0.998 to 1.043)	0.071
	3	1.078	1.027 to 1.132	0.002	1.056	(1.034 to 1.078)	<0.001
	2	1.145	1.093 to 1.2	<0.001	1.078	(1.057 to 1.1)	<0.001
	1	1.202	1.148 to 1.259	<0.001	1.153	(1.131 to 1.176)	<0.001
**Origin**	(Domicile is reference)	1			1		
	Other	1.381	1.297 to 1.471	<0.001	1.452	(1.409 to 1.496)	<0.001
	Transfer	1.493	1.342 to 1.662	<0.001	1.651	(1.567 to 1.739)	<0.001
**Diagnosis Category**	High risk (Low risk is reference)	3.476	3.368 to 3.587	<0.001	1.729	(1.7 to 1.759)	<0.001
**Treatment Category**	Non-operative (reference)	1			1		
	Operative laparotomy	1.202	1.106 to 1.306	<0.001	1.083	(1.032 to 1.136)	0.001
	Operative laparoscopy	0.124	0.082 to 0.188	<0.001	0.319	(0.293 to 0.348)	<0.001
	Operative GI other	0.456	0.438 to 0.475	<0.001	0.988	(0.973 to 1.004)	0.132
	Operative skin/soft tissue	0.503	0.468 to 0.541	<0.001	0.77	(0.748 to 0.791)	<0.001
	Operative other non-GI	0.841	0.812 to 0.87	<0.001	0.95	(0.935 to 0.965)	<0.001
**Time**	Admission year	0.926	0.923 to 0.928	<0.001	0.955	(0.954 to 0.956)	<0.001

OR, odds ratio; SURC, Scottish Urban Rural Classification; CCI, Charlson co-morbidity index; SIMD; Scottish index of multiple deprivation (1, most deprived; 5, least deprived); GI, gastrointestinal. Model summary: Cox–Snell *R*^2^ = 0.038; Nagelkerke *R*^2^ = 0.255. Number of cases/observations entered into the model: 1 564 629.

### Rurality and mortality

Patients who lived in the most rural category (SURC 6) had higher odds of survival than those in the most urban category (SURC 1) (OR 0.800, 95 per cent c.i. 0.755 to 0.848) (*Table 4*). With 1-year mortality as the dependent variable, those in the most rural category (SURC 6) still had higher odds of survival than those in the most urban category (OR 0.930, 95 per cent c.i. 0.908 to 0.954) (*Table 4*). Sensitivity analyses of three-fold SURC categories also demonstrated improved odds of survival in the most rural category (category 3) compared with the most urban category, regarding inpatient mortality (OR 0.821, 95 per cent c.i. 0.777 to 0.866) and 1-year mortality (OR 0.910, 95 per cent c.i. 0.889 to 0.931; *Table 5*). Similarly, evaluating two-fold SURC categories showed a survival advantage with the most rural category (category 2) compared with the most urban category, evaluating inpatient mortality (OR 0.870, 95 per cent c.i. 0.837 to 0.904), and 1-year mortality (OR 0.933, 95 per cent c.i. 0.918 to 0.949; *Table 6*).

**Table 4:** Multiple logistic regression: inpatient and 1-year mortality as dependent variable, with six-fold Scottish urban/rural classification as covariate of interest, for all admissions

		Inpatient mortality as dependent			1-year mortality as dependent		
		OR	95% c.i.	*P*	OR	95% c.i.	*P*
**Six-fold SURC**	1 (reference)	1			1		
	2	0.999	(0.969 to 1.031)	0.973	1.055	(1.04 to 1.069)	<0.001
	3	0.952	(0.904 to 1.004)	0.068	1.023	(1 to 1.046)	0.055
	4	0.814	(0.761 to 0.87)	<0.001	0.941	(0.914 to 0.968)	<0.001
	5	0.89	(0.845 to 0.938)	<0.001	0.973	(0.952 to 0.995)	0.018
	6	0.8	(0.755 to 0.848)	<0.001	0.93	(0.908 to 0.954)	<0.001
**Age category**	16–30 years (reference)	1			1		
	31–45 years	2.88	(2.297 to 3.612)	<0.001	3.221	(3.042 to 3.41)	<0.001
	46–60 years	9.039	(7.32 to 11.161)	<0.001	7.498	(7.103 to 7.915)	<0.001
	61–75 years	21.564	(17.508 to 26.559)	<0.001	15.214	(14.422 to 16.048)	<0.001
	More than 75 years	50.338	(40.89 to 61.969)	<0.001	32.564	(30.873 to 34.348)	<0.001
**Sex**	Female (male is reference)	1.123	(1.094 to 1.153)	<0.001	0.892	(0.882 to 0.902)	<0.001
**CCI 10 year**	0; no co-morbidity (reference)	1			1		
	1–2; mild co-morbidity	3.487	(3.325 to 3.658)	<0.001	3.363	(3.308 to 3.419)	<0.001
	3–4; moderate co-morbidity	5.81	(5.519 to 6.117)	<0.001	6.177	(6.061 to 6.295)	<0.001
	>4; severe co-morbidity	14.025	(13.37 to 14.712)	<0.001	22.825	(22.409 to 23.249)	<0.001
**SIMD quintile**	5 (reference)	1			1		
	4	1.116	(1.06 to 1.175)	<0.001	1.03	(1.007 to 1.053)	0.009
	3	1.1	(1.046 to 1.155)	<0.001	1.061	(1.039 to 1.084)	<0.001
	2	1.154	(1.101 to 1.209)	<0.001	1.075	(1.053 to 1.097)	<0.001
	1	1.212	(1.157 to 1.269)	<0.001	1.157	(1.134 to 1.18)	<0.001
**Origin**	(Domicile is reference)	1			1		
	Other	1.375	(1.291 to 1.464)	<0.001	1.445	(1.402 to 1.489)	<0.001
	Transfer	1.45	(1.303 to 1.614)	<0.001	1.618	(1.536 to 1.705)	<0.001
**Diagnosis category**	High risk (Low risk is reference)	3.453	(3.346 to 3.563)	<0.001	1.723	(1.694 to 1.753)	<0.001
**Treatment category**	Non-operative (reference)	1			1		
	Operative laparotomy	1.202	(1.106 to 1.307)	<0.001	1.081	(1.03 to 1.134)	0.001
	Operative laparoscopy	0.123	(0.082 to 0.186)	<0.001	0.317	(0.291 to 0.346)	<0.001
	Operative GI other	0.452	(0.434 to 0.471)	<0.001	0.984	(0.969 to 0.999)	0.036
	Operative skin/soft tissue	0.502	(0.466 to 0.539)	<0.001	0.768	(0.747 to 0.789)	<0.001
	Operative other non-GI	0.842	(0.813 to 0.871)	<0.001	0.95	(0.935 to 0.965)	<0.001
**Time**	Admission year	0.925	(0.923 to 0.927)	<0.001	0.955	(0.954 to 0.956)	<0.001

OR, odds ratio; SURC, Scottish Urban Rural Classification; CCI, Charlson co-morbidity index; SIMD, Scottish index of multiple deprivation (1, most deprived; 5, least deprived); GI, gastrointestinal. Model summary: Cox–Snell *R*^2^ = 0.038; Nagelkerke *R*^2^ = 0.254. Number of cases/observations entered into the model: 1 564 629.

**Table 5:** Multiple logistic regression (sensitivity analysis): inpatient and 1-year mortality as dependent variable, with three-fold Scottish urban/rural classification as covariate of interest

		Inpatient mortality as dependent			1-year mortality as dependent		
		OR	95% c.i.	*P*	OR	95% c.i.	*P*
**Three-fold SURC**	1 (reference)	1			1		
	2	0.911	(0.868 to 0.956)	<0.001	0.952	(0.933 to 0.972)	<0.001
	3	0.821	(0.777 to 0.866)	<0.001	0.91	(0.889 to 0.931)	<0.001
**Age category**	16–30 years (reference)	1			1		
	31–45 years	2.882	(2.298 to 3.614)	<0.001	3.222	(3.043 to 3.412)	<0.001
	46–60 years	9.032	(7.315 to 11.152)	<0.001	7.5	(7.105 to 7.917)	<0.001
	61–75 years	21.526	(17.477 to 26.513)	<0.001	15.218	(14.426 to 16.053)	<0.001
	More than 75 years	50.184	(40.765 to 61.78)	<0.001	32.529	(30.839 to 34.311)	<0.001
**Sex**	Female (male is reference)	1.123	(1.094 to 1.153)	<0.001	0.892	(0.882 to 0.902)	<0.001
**CCI 10 year**	0; no co-morbidity (reference)	1			1		
	1–2; mild co-morbidity	3.489	(3.326 to 3.659)	<0.001	3.363	(3.309 to 3.419)	<0.001
	3–4; moderate co-morbidity	5.814	(5.522 to 6.121)	<0.001	6.176	(6.06 to 6.295)	<0.001
	>4; severe co-morbidity	14.036	(13.381 to 14.724)	<0.001	22.83	(22.414 to 23.255)	<0.001
**SIMD quintile**	5 (reference)	1			1		
	4	1.217	(1.163 to 1.274)	<0.001	1.158	(1.135 to 1.181)	<0.001
	3	1.145	(1.093 to 1.2)	<0.001	1.08	(1.058 to 1.102)	<0.001
	2	1.084	(1.032 to 1.139)	0.001	1.063	(1.04 to 1.085)	<0.001
	1	1.101	(1.046 to 1.159)	<0.001	1.03	(1.007 to 1.053)	0.01
**Origin**	(Domicile is reference)	1			1		
	Other	1.363	(1.28 to 1.452)	<0.001	1.443	(1.4 to 1.487)	<0.001
	Transfer	1.436	(1.29 to 1.597)	<0.001	1.623	(1.541 to 1.71)	<0.001
**Diagnosis category**	High risk (Low risk is reference)	3.457	(3.35 to 3.567)	<0.001	1.725	(1.696 to 1.755)	<0.001
**Treatment category**	Non-operative (reference)	1			1		
	Operative laparotomy	1.195	(1.1 to 1.299)	<0.001	1.081	(1.03 to 1.134)	0.002
	Operative laparoscopy	0.123	(0.082 to 0.186)	<0.001	0.318	(0.291 to 0.346)	<0.001
	Operative GI other	0.452	(0.434 to 0.471)	<0.001	0.985	(0.97 to 1)	0.053
	Operative skin/soft tissue	0.501	(0.466 to 0.538)	<0.001	0.768	(0.747 to 0.79)	<0.001
	Operative other non-GI	0.838	(0.809 to 0.867)	<0.001	0.949	(0.934 to 0.964)	<0.001
**Time**	Admission year	0.925	(0.923 to 0.927)	<0.001	0.955	(0.954 to 0.956)	<0.001

OR, odds ratio; SURC, Scottish Urban Rural Classification; CCI, Charlson co-morbidity index; SIMD, Scottish index of multiple deprivation (1, most deprived; 5, least deprived); GI, gastrointestinal. Model summary: Cox–Snell *R*^2^ = 0.038; Nagelkerke *R*^2^ = 0.254. Number of cases/observations entered into the model: 1 564 629.

**Table 6:** Multiple logistic regression (sensitivity analysis): inpatient and 1-year mortality as dependent variable, with two-fold Scottish urban/rural classification as covariate of interest

		Inpatient mortality as dependent			1-year mortality as dependent		
		OR	95% c.i.	*P*	OR	95% c.i.	*P*
**Two-fold SURC**	2 (1 is reference)	0.87	(0.837 to 0.904)	<0.001	0.933	(0.918 to 0.949)	<0.001
**Age category**	16–30 years (reference)	1			1		
	31–45 years	2.882	(2.298 to 3.615)	<0.001	3.222	(3.043 to 3.412)	<0.001
	46–60 years	9.031	(7.314 to 11.151)	<0.001	7.5	(7.105 to 7.917)	<0.001
	61–75 years	21.513	(17.467 to 26.497)	<0.001	15.213	(14.422 to 16.048)	<0.001
	More than 75 years	50.117	(40.71 to 61.697)	<0.001	32.507	(30.819 to 34.288)	<0.001
**Sex**	Female (male is reference)	1.123	(1.094 to 1.153)	<0.001	0.892	(0.882 to 0.902)	<0.001
**CCI 10 year**	0; no co-morbidity (reference)	1			1		
	1–2; mild co-morbidity	3.488	(3.326 to 3.658)	<0.001	3.363	(3.308 to 3.419)	<0.001
	3–4; moderate co-morbidity	5.813	(5.522 to 6.12)	<0.001	6.176	(6.059 to 6.294)	<0.001
	>4; severe co-morbidity	14.037	(13.382 to 14.725)	<0.001	22.83	(22.414 to 23.255)	<0.001
**SIMD quintile**	5 (reference)	1			1		
	4	1.101	(1.046 to 1.159)	<0.001	1.029	(1.007 to 1.053)	0.011
	3	1.078	(1.027 to 1.132)	0.003	1.06	(1.038 to 1.082)	<0.001
	2	1.143	(1.091 to 1.197)	<0.001	1.079	(1.057 to 1.101)	<0.001
	1	1.215	(1.161 to 1.272)	<0.001	1.157	(1.134 to 1.18)	<0.001
**Origin**	(Domicile is reference)	1			1		
	Other	1.36	(1.277 to 1.448)	<0.001	1.441	(1.399 to 1.485)	<0.001
	Transfer	1.428	(1.283 to 1.588)	<0.001	1.62	(1.538 to 1.707)	<0.001
**Diagnosis category**	High risk (Low risk is reference)	3.46	(3.353 to 3.57)	<0.001	1.726	(1.697 to 1.756)	<0.001
**Treatment category**	Non-operative (reference)	1			1		
	Operative laparotomy	1.195	(1.099 to 1.298)	<0.001	1.08	(1.03 to 1.133)	0.002
	Operative laparoscopy	0.123	(0.082 to 0.186)	<0.001	0.318	(0.292 to 0.347)	<0.001
	Operative GI other	0.452	(0.434 to 0.471)	<0.001	0.985	(0.97 to 1)	0.054
	Operative skin/soft tissue	0.5	(0.465 to 0.538)	<0.001	0.768	(0.747 to 0.79)	<0.001
	Operative other non-GI	0.836	(0.808 to 0.866)	<0.001	0.948	(0.933 to 0.963)	<0.001
**Time**	Admission year	0.925	(0.923 to 0.927)	<0.001	0.955	(0.954 to 0.956)	<0.001

OR, odds ratio; SURC, Scottish Urban Rural Classification; CCI, Charlson co-morbidity index; SIMD, Scottish index of multiple deprivation (1, most deprived, 5, least deprived); GI, gastrointestinal. Model summary: Cox–Snell *R*^2^ = 0.038; Nagelkerke *R*^2^ = 0.254. Number of cases/observations entered into the model: 1 564 629.

### Subgroup analyses

#### Patients who required operative treatment

A total of 663 586 patients had an operation. Compared with those closest to the hospital, those living in the farthest quartile had lower odds of in-hospital mortality (OR 0.827, 95 per cent c.i. 0.780 to 0.877), and 1-year mortality (OR 0.910, 95 per cent c.i. 0.888 to 0.932; *Table S3*). Those admitted from the most rural category (SURC 6) also had reduced odds of inpatient mortality (OR 0.889, 95 per cent c.i. 0.816 to 0.968) compared with the most urban category. However, there was no difference in 1-year mortality (OR 0.978, 95 per cent c.i. 0.944 to 1.013; *Table S4*).

#### Patients who required emergency laparotomy

Subgroup analysis, including only those who underwent an emergency laparotomy (*n* = 20 669), showed no significant difference of inpatient or 1-year mortality either in distance from hospital (*Table S5*), or rurality (*Table S6*).

#### Patients who were transferred

Analysis of those who were transferred between hospitals (*n* = 11 869) showed increased mortality with increased distance from hospital and increased rurality (*Tables S7* and *S8*). There were significant increases of inpatient mortality in the third distance quartile (OR 1.520, 95 per cent c.i. 1.019 to 2.266), and 1-year mortality in the third and fourth distance quartiles (OR 1.283, 95 per cent c.i. 1.053 to 1.564; OR 1.259, 95 per cent c.i. 1.059 to 1.497 respectively). There were also significant increases in the odds of 1-year mortality in SURC 3, 4, and 5 (OR 1.272, 95 per cent c.i. 1.016 to 1.592; OR 1.287, 95 per cent c.i. 1.02 to 1.624; OR 1.250, 95 per cent c.i. 1.01 to 1.546 respectively).

## Discussion

For EGS patients, including those who were managed non-operatively, there seems to be increased survival for those residing further from the admitting hospital, and/or in more remote locations. This paradoxical finding may be explained by the types of patients who are admitted under general surgical care in the UK, many of whom suffer from low-acuity conditions. This beneficial effect is no longer apparent when patients with more serious illness—such as those requiring emergency laparotomy—are considered, or those patients who are transferred between hospitals because they require a high level of care. However, it is reassuring to know that patients who reside in remote and rural areas and require emergency laparotomy do not have worse mortality than those who live in more central locations.

The evidence from similar published literature regarding whether rurality or distance from hospital affects mortality is inconsistent, ranging from beneficial to detrimental. A UK study that evaluated patients included in the National Emergency Laparotomy Audit from 2013–2016 showed that the estimated travel time between home and hospital was not a primary determinant of short-term mortality^1^. The rural emergency laparotomy audit in Australia reported similar findings^4^. In the USA, the occurrence of adverse postoperative events after EGS was not related to whether patients lived in rural areas^3^. Other studies confirm the safety of undertaking emergency abdominal surgery in non-urban centres^5,6^. A Scottish study found that distance from hospital had no significant impact on community mortality rates of ruptured abdominal aortic aneurysms^8^. More recently a study demonstrated that increased distance to hospital led to decreased mortality after open repair of ruptured abdominal aortic aneurysm between 1990–2011; however, the authors suggested that this could have been due to survivor bias^9^. In fields related to EGS, the evidence is similarly inconsistent. In trauma care, several US studies have shown increased mortality risk for rural trauma populations^26–29^. However, a large US study found that mortality did not differ between rural and urban regions, even though a higher proportion of rural deaths occurred within 24 h compared with urban deaths^30^. In Scotland, long prehospital times in rural environments did not affect mortality in moderately and severely injured patients^31^.

There are several possible explanations for the apparent benefit seen in the study population. First, patients who did not survive to hospital admission were not included. It is possible that a greater proportion of patients from longer distances, or more remote areas, did not survive the journey to hospital, and therefore their exclusion could have reduced mortality for these subgroups and biased the results. However, deaths during transfer are rare in Scotland^32,33^. Second, there could be rural or distance bias in patient referrals to EGS care. Perhaps rural general practitioners (GPs) have a lower threshold for sending patients for assessment to hospitals as EGS admissions, thus patients may be less unwell than urban EGS patients, accounting for lower mortality rates. However, a recent survey of GPs from 20 European countries identified that rural GPs are just as likely to refer patients for specialist care as urban GPs^34^. Similarly, surgeons receiving referrals from rural or longer distance areas, may be more willing to accept the patient under their care, because of the lack of alternative resource and the medical consequences of not addressing major pathology that presents with mild symptoms. Third, there may exist unknown confounders that provide survival benefit to those living in rural locations, or locations far from major hospitals. Perhaps certain lifestyle factors specific to rural areas better prepare patients for EGS admissions, which improves their odds of survival, such as levels of physical activity^35–37^. There is a complex relationship between rurality, socioeconomic status, and physical activity, such that as remoteness and socioeconomic status increased, physical activity increased in Australia^38^. Although adjusted for in modelling, the data in this study demonstrate that a high proportion of Scottish urban dwellers (SURC 1 and 2) live in low-deprivation areas (SIMD 1 and 2), whereas a comparatively small proportion of rural dwellers (SURC 5 and 6) live in low-deprivation areas (*Table 2*). These data demonstrating that rural survival is higher may be because rural dwellers represent a less-deprived cohort, which cannot be ruled out completely.

This study has limitations. There are several variables that are not accounted for in the data, which would have provided a more thorough risk adjustment, relating to physiological, or biochemical parameters. Transfer information did not include the specific hospital or level of hospital that patients were transferred from, precluding a more detailed analysis. Both rurality and distance from hospital were analysed, which are related but different. For example, a patient living in a rural location may live very close to their admitting hospital (rural, but short travel time); however, such hospitals are usually smaller with fewer facilities. Conversely, a patient may be living in a less rural location, but their closest admitting hospital may be 20 km away (not rural, but long travel time). Adjusting for hospital type and volume was considered, but there was collinearity between these variables and rurality, so they were excluded from the analyses; however, a recently published paper addressed the impact of hospital and surgeon admission volume, and mortality^39^. If distance from hospital was associated with outcome, one would expect this association to be linear, but those in the third quartile did not have better survival. This may reflect other hidden confounders. Finally, the event (mortality) rates were very low (1–2 per cent), which would normally affect the performance of logistic regression models, but because of the large sample size the analyses are still valid.

This study also has many strengths, the most important being its size, and the quality, and consistency of the data. Scotland’s population-based health data are regularly audited for accuracy and include a validated urban/rural classification that facilitates studies of this kind^40^. These findings have important health policy implications. Rural surgery is an important part of healthcare provision in geographically dispersed populations^41–43^. Access to surgical services, especially out of hours, can be limited for those in remote and rural areas^44–46^. This is becoming increasingly problematic in some areas, including parts of the USA, where the rate of rural hospital closures has risen in the past decade, largely due to financial, market, and staffing issues^45^. Centralization of health services is an obvious but contentious solution, given the cost and inconvenience of travelling large distances from patients’ home to receive care^47^, and the results do not support this strategy. The Scottish Government published a report stating the role of rural general hospitals for EGS patients: ‘24-hour surgical services should provide local assessment, triage, resuscitation stabilization of emergency surgical and trauma patients followed by admission and surgical intervention, if appropriate, and transfer, when necessary, in collaboration with the relevant receiving hospital’^48^. Clearly, this paper addresses a topic of great importance to the Scottish health system, for which policies and procedures have been intentionally designed to avoid further centralization by providing adequate rural care and allowing transfer when necessary.

A key area of future research is the early identification and prognostication of patients at high risk of requiring transfer to higher levels of care from rural and distant populations. Several clinical decision support tools that predict mortality and need for intensive care have been developed^49,50^. Trauma systems have widely adopted trauma field triage decision tools to decide whether to bypass smaller trauma units and convey to large trauma centres^51,52^. An analogous system devised for EGS patients who may require transfer to centres with specialist surgical services or ICUs, may improve care for rural and distant populations^53^.

## Supplementary Material

zrac032_Supplementary_Data

## Data Availability

Study data, analytic methods, and study materials were hosted on the National Safe Haven, which the authors no longer have access to. Requests can be made to the eDRIS Team (Public Health Scotland) with reference to PBPP 1819-0340.
