# Supplementary material for: Emergency general surgery: impact of distance and rurality on mortality
Source: BJS Open. 2022 Apr 25;6(2):zrac032. doi: 10.1093/bjsopen/zrac032 (PMC9035437; doi:10.1093/bjsopen/zrac032)
Supplement: zrac032_Supplementary_Data [file zrac032_supplementary_data.docx]

Table S1. Reproduced from Symons, *et al*,^15^ (Table S1) describing ‘high-risk’ ICD-10 diagnostic codes.

| **ICD-10 code and diagnosis** | **No. of patients** | **30-day in-hospital mortality (%)** |
| --- | --- | --- |
| **Gastrointestinal ulcers** |  |  |
| K25.1 Gastric ulcer, acute with perforation | 1254 | 23.6 |
| K25.2 Gastric ulcer, acute with both haemorrhage and perforation | 166 | 32.5 |
| K25.5 Gastric ulcer, chronic or unspecified with perforation | 3995 | 19.9 |
| K25.6 Gastric ulcer, chronic or unspecified with both haemorrhage and perforation | 373 | 31.1 |
| K26.1 Duodenal ulcer, acute with perforation | 3815 | 19.3 |
| K26.2 Duodenal ulcer, acute with both haemorrhage and perforation | 470 | 31.1 |
| K26.5 Duodenal ulcer, chronic or unspecified with perforation | 13 397 | 19.8 |
| K26.6 Duodenal ulcer, chronic or unspecified with both haemorrhage and perforation | 1156 | 30.6 |
| K27.1 Peptic ulcer, acute with perforation | 189 | 39.2 |
| K27.2 Peptic ulcer, acute with both haemorrhage and perforation | 24 | 58.3 |
| K27.5 Peptic ulcer, chronic or unspecified with perforation | 687 | 37.4 |
| K27.6 Peptic ulcer, chronic or unspecified with both haemorrhage and perforation | 83 | 50.6 |
| K28.0 Gastrojejunal ulcer, acute with haemorrhage | 37 | 5.4 |
| K28.1 Gastrojejunal ulcer, acute with perforation | 23 | 13.0 |
| K28.2 Gastrojejunal ulcer, acute with both haemorrhage and perforation | 9 | 33.3 |
| K28.3 Gastrojejunal ulcer, acute without haemorrhage or perforation | 18 | 11.1 |
| K28.5 Gastrojejunal ulcer, chronic or unspecified with perforation | 99 | 21.2 |
| K28.6 Gastrojejunal ulcer, chronic or unspecified with both haemorrhage and perforation | 10 | 60.0 |
| K28.7 Gastrojejunal ulcer, chronic without haemorrhage or perforation | 25 | 12.0 |
| K28.9 Gastrojejunal ulcer, unspecified without haemorrhage or perforation | 220 | 5.0 |
| **Hernias** |  |  |
| K40.0 Bilateral inguinal hernia with obstruction without gangrene | 736 | 6.8 |
| K40.1 Bilateral inguinal hernia, with gangrene | 44 | 13.6 |
| K40.4 Unilateral or unspecified inguinal hernia, with gangrene | 604 | 13.7 |
| K41.0 Bilateral femoral hernia, with obstruction, without gangrene | 253 | 10.7 |
| K41.1 Bilateral femoral hernia, with gangrene | 40 | 22.5 |
| K41.3 Unilateral or unspecified femoral hernia with obstruction without gangrene | 11 584 | 8.2 |
| K41.4 Unilateral or unspecified femoral hernia, with gangrene | 1123 | 11.9 |
| K42.1 Umbilical hernia with gangrene | 759 | 7.9 |
| K43.0 Ventral hernia with obstruction, without gangrene | 11 518 | 5.7 |
| K43.1 Ventral hernia with gangrene | 592 | 15.2 |
| K44.0 Diaphragmatic hernia with obstruction, without gangrene | 1247 | 11.3 |
| K44.1 Diaphragmatic hernia with gangrene | 141 | 12.8 |
| K45.0 Other specified abdominal hernia with obstruction without gangrene | 1280 | 12.6 |
| K45.1 Other specified abdominal hernia with gangrene | 141 | 17.0 |
| K46.0 Unspecified abdominal hernia with obstruction without gangrene | 1006 | 12.7 |
| K46.1 Unspecified abdominal hernia with gangrene | 88 | 12.5 |
| **Bowel ischaemia** |  |  |
| K55.0 Acute vascular disorders of intestine | 12 919 | 49.4 |
| K55.1 Chronic vascular disorders of intestine | 892 | 30.8 |
| K55.8 Other vascular disorders of intestine | 261 | 16.5 |
| K55.9 Vascular disorder of intestine, unspecified | 6694 | 46.8 |
| **Bowel obstruction** |  |  |
| K56.0 Paralytic ileus | 995 | 14.3 |
| K56.1 Intussusception | 837 | 5.1 |
| K56.2 Volvulus | 20 726 | 10.6 |
| K56.3 Gallstone ileus | 1888 | 10.2 |
| K56.4 Other impaction of intestine | 7950 | 5.2 |
| K56.5 Intestinal adhesions [bands] with obstruction | 34 944 | 5.5 |
| K56.6 Other and unspecified intestinal obstruction | 90 048 | 11.8 |
| K56.7 Ileus, unspecified | 1264 | 7.3 |
| **Diverticulitis** |  |  |
| K57.0 Diverticular disease of small intestine with perforation and abscess | 1170 | 17.5 |
| K57.2 Diverticular disease of large intestine with perforation and abscess | 17 593 | 16.5 |
| K57.4 Diverticular disease of both small and large intestine with perforation + abscess | 781 | 18.8 |
| K57.8 Diverticular disease of intestine, part unspecified, with perforation and abscess | 5956 | 25.1 |
| **Disorders of peritoneum** |  |  |
| K65.0 Acute peritonitis | 13 311 | 20.7 |
| K65.8 Other peritonitis | 2421 | 18.9 |
| K65.9 Peritonitis, unspecified | 9949 | 41.9 |
| K66.1 Haemoperitoneum | 1550 | 15.6 |
| K66.8 Other specified disorders of peritoneum | 836 | 7.8 |
| K66.9 Disorder of peritoneum, unspecified | 151 | 11.3 |
| **Liver and biliary conditions** |  |  |
| K76.2 Central haemorrhagic necrosis of liver | 5 | 20.0 |
| K76.3 Infarction of liver | 55 | 49.1 |
| K76.8 Other specified diseases of liver | 2881 | 6.5 |
| K80.3 Calculus of bile duct with cholangitis | 8872 | 5.4 |
| K82.0 Obstruction of gallbladder | 417 | 5.8 |
| K82.2 Perforation of gallbladder | 1534 | 17.4 |
| K82.3 Fistula of gallbladder | 132 | 10.6 |
| K83.0 Cholangitis | 14 271 | 8.5 |
| K83.1 Obstruction of bile duct | 21 360 | 6.8 |
| K83.2 Perforation of bile duct | 84 | 9.5 |
| **Miscellaneous diagnoses** |  |  |
| K22.3 Perforation of oesophagus | 1008 | 26.4 |
| K31.0 Acute dilatation of stomach | 181 | 10.5 |
| K31.1 Adult hypertrophic pyloric stenosis | 2595 | 10.3 |
| K31.5 Obstruction of duodenum | 1177 | 7.6 |
| K31.6 Fistula of stomach and duodenum | 158 | 7.6 |
| K59.3 Megacolon, not elsewhere classified | 1916 | 13.2 |
| K59.8 Other specified functional intestinal disorders | 3106 | 7.7 |
| K63.0 Abscess of intestine | 1384 | 7.2 |
| K63.1 Perforation of intestine (non-traumatic) | 13 872 | 46.2 |
| K63.4 Enteroptosis | 144 | 5.6 |
| K63.8 Other specified diseases of intestine | 2302 | 6.1 |
| **Total** | **367 796** |  |

ICD-10, International Classification of Diseases, tenth revision.

Table S2: Scottish Government Urban Rural Classification, 6-fold (SURC).^23^

| Class | Class Name | Description |
| --- | --- | --- |
| 1 | Large Urban Areas | Settlements of 125,000 people and over. |
| 2 | Other Urban Areas | Settlements of 10,000 to 124,999 people. |
| 3 | Accessible Small Towns | Settlements of 3,000 to 9,999 people, and within a 30 minute drive time of a Settlement of 10,000 or more. |
| 4 | Remote Small Towns | Settlements of 3,000 to 9,999 people, and with a drive time of over 30 minutes to a Settlement of 10,000 or more. |
| 5 | Accessible Rural Areas | Areas with a population of less than 3,000 people, and within a 30 minute drive time of a Settlement of 10,000 or more. |
| 6 | Remote Rural Areas | Areas with a population of less than 3,000 people, and with a drive time of over 30 minutes to a Settlement of 10,000 or more. |

Table S3: Subgroup Analysis: Excluding Non-operative admissions. Multiple logistic regression: Inpatient and 1-year mortality as dependent variable, with distance from hospital as covariate of interest.

|  |  | Inpatient Mortality as dependent | | | 1-year Mortality as dependent | | |
| --- | --- | --- | --- | --- | --- | --- | --- |
|  |  | OR | 95% CI | p | OR | 95% CI | p |
| Distance from Hospital (km) | 0 - 2.9km (reference) | 1 |  |  | 1 |  |  |
|  | 2.9 - 6.4km | 0.962 | (0.907 to 1.02) | 0.198 | 0.975 | (0.95 to 0.999) | 0.044 |
|  | 6.4 - 15.2km | 1.005 | (0.948 to 1.066) | 0.868 | 1.021 | (0.996 to 1.047) | 0.108 |
|  | >15.2km | 0.827 | (0.78 to 0.877) | <0.001 | 0.91 | (0.888 to 0.932) | <0.001 |
| Age category | 16-30 (reference) | 1 |  |  | 1 |  |  |
|  | 31-45 | 2.265 | (1.636 to 3.135) | <0.001 | 3.507 | (3.192 to 3.853) | <0.001 |
|  | 46-60 | 7.16 | (5.313 to 9.648) | <0.001 | 8.756 | (8.008 to 9.573) | <0.001 |
|  | 61-75 | 16.116 | (12.011 to 21.625) | <0.001 | 17.236 | (15.78 to 18.826) | <0.001 |
|  | >75 | 35.323 | (26.342 to 47.366) | <0.001 | 35.825 | (32.8 to 39.128) | <0.001 |
| Sex | Female (Male is reference) | 1.125 | (1.079 to 1.173) | <0.001 | 0.912 | (0.896 to 0.928) | <0.001 |
| CCI 10 year | 0; no comorbidity (reference) | 1 |  |  | 1 |  |  |
|  | 1-2; mild comorbidity | 3.494 | (3.239 to 3.77) | <0.001 | 3.508 | (3.422 to 3.597) | <0.001 |
|  | 3-4; moderate comorbidity | 5.806 | (5.345 to 6.307) | <0.001 | 6.494 | (6.307 to 6.687) | <0.001 |
|  | >4; severe comorbidity | 14.732 | (13.657 to 15.891) | <0.001 | 24.678 | (23.994 to 25.383) | <0.001 |
| SIMD Quintile | 5 (reference) | 1 |  |  | 1 |  |  |
|  | 4 | 1.107 | (1.023 to 1.199) | 0.012 | 1.027 | (0.994 to 1.061) | 0.107 |
|  | 3 | 1.091 | (1.01 to 1.178) | 0.026 | 1.074 | (1.041 to 1.108) | <0.001 |
|  | 2 | 1.19 | (1.105 to 1.281) | <0.001 | 1.114 | (1.081 to 1.148) | <0.001 |
|  | 1 | 1.283 | (1.192 to 1.38) | <0.001 | 1.183 | (1.148 to 1.219) | <0.001 |
| Origin | (Domicile is reference) | 1 |  |  | 1 |  |  |
|  | Other | 1.384 | (1.25 to 1.532) | <0.001 | 1.514 | (1.445 to 1.585) | <0.001 |
|  | Transfer | 1.394 | (1.186 to 1.637) | <0.001 | 1.682 | (1.564 to 1.809) | <0.001 |
| Diagnosis Category | High risk (Low Risk is reference) | 2.339 | (2.224 to 2.459) | <0.001 | 1.521 | (1.485 to 1.558) | <0.001 |
| Treatment Category | Operative- GI- other (reference) | 1 |  |  | 1 |  |  |
|  | Operative- laparotomy | 2.864 | (2.622 to 3.128) | <0.001 | 1.148 | (1.094 to 1.206) | <0.001 |
|  | Operative- laparoscopy | 0.275 | (0.182 to 0.415) | <0.001 | 0.305 | (0.28 to 0.333) | <0.001 |
|  | Operative- skin/soft tissue | 1.088 | (1.006 to 1.176) | 0.036 | 0.773 | (0.75 to 0.796) | <0.001 |
|  | Operative- other non-GI | 1.67 | (1.593 to 1.751) | <0.001 | 0.957 | (0.939 to 0.976) | <0.001 |
| Time | Admission Year | 0.918 | (0.914 to 0.922) | <0.001 | 0.964 | (0.962 to 0.965) | <0.001 |
| OR= odds ratio; CI= confidence interval; SURC= Scottish Urban/Rural Classification; CCI= Charlson Comorbidity Index; SIMD= Scottish Index of Multiple Deprivation (1=most deprived; 5=least deprived); GI= gastrointestinal. Model Summary: Cox & Snell R Square=0.034; Nagelkerke R Square=0.241. Number of cases/observations entered into the model: 663586 | | | | | | | |

Table S4: Subgroup Analysis: Excluding Non-operative admissions. Multiple logistic regression: Inpatient and 1-year mortality as dependent variable, with 6-Fold SURC as covariate of interest.

|  |  | Inpatient Mortality as dependent | | | 1-year Mortality as dependent | | |
| --- | --- | --- | --- | --- | --- | --- | --- |
|  |  | OR | 95% CI | p | OR | 95% CI | p |
| 6-fold SURC | 1 (reference) | 1 |  |  | 1 |  |  |
|  | 2 | 1.001 | (0.952 to 1.053) | 0.97 | 1.086 | (1.063 to 1.109) | <0.001 |
|  | 3 | 0.933 | (0.858 to 1.014) | 0.104 | 1.059 | (1.023 to 1.096) | 0.001 |
|  | 4 | 0.943 | (0.855 to 1.039) | 0.236 | 0.977 | (0.937 to 1.018) | 0.264 |
|  | 5 | 0.922 | (0.85 to 1) | 0.05 | 0.984 | (0.952 to 1.017) | 0.344 |
|  | 6 | 0.889 | (0.816 to 0.968) | 0.007 | 0.978 | (0.944 to 1.013) | 0.217 |
| Age category | 16-30 (reference) | 1 |  |  | 1 |  |  |
|  | 31-45 | 2.264 | (1.636 to 3.134) | <0.001 | 3.505 | (3.19 to 3.851) | <0.001 |
|  | 46-60 | 7.165 | (5.317 to 9.655) | <0.001 | 8.753 | (8.005 to 9.57) | <0.001 |
|  | 61-75 | 16.167 | (12.049 to 21.693) | <0.001 | 17.235 | (15.779 to 18.825) | <0.001 |
|  | >75 | 35.47 | (26.452 to 47.564) | <0.001 | 35.87 | (32.841 to 39.178) | <0.001 |
| Sex | Female (Male is reference) | 1.125 | (1.079 to 1.173) | <0.001 | 0.912 | (0.896 to 0.928) | <0.001 |
| CCI 10 year | 0; no comorbidity (reference) | 1 |  |  | 1 |  |  |
|  | 1-2; mild comorbidity | 3.486 | (3.232 to 3.76) | <0.001 | 3.502 | (3.415 to 3.59) | <0.001 |
|  | 3-4; moderate comorbidity | 5.78 | (5.321 to 6.279) | <0.001 | 6.474 | (6.288 to 6.666) | <0.001 |
|  | >4; severe comorbidity | 14.655 | (13.586 to 15.807) | <0.001 | 24.568 | (23.887 to 25.268) | <0.001 |
| SIMD Quintile | 5 (reference) | 1 |  |  | 1 |  |  |
|  | 4 | 1.111 | (1.024 to 1.205) | 0.011 | 1.03 | (0.997 to 1.065) | 0.079 |
|  | 3 | 1.088 | (1.006 to 1.177) | 0.035 | 1.067 | (1.034 to 1.102) | <0.001 |
|  | 2 | 1.186 | (1.101 to 1.278) | <0.001 | 1.103 | (1.07 to 1.137) | <0.001 |
|  | 1 | 1.288 | (1.197 to 1.386) | <0.001 | 1.185 | (1.15 to 1.222) | <0.001 |
| Origin | (Domicile is reference) | 1 |  |  | 1 |  |  |
|  | Other | 1.364 | (1.232 to 1.51) | <0.001 | 1.497 | (1.43 to 1.567) | <0.001 |
|  | Transfer | 1.325 | (1.129 to 1.555) | 0.001 | 1.629 | (1.514 to 1.751) | <0.001 |
| Diagnosis Category | High risk (Low Risk is reference) | 2.328 | (2.214 to 2.448) | <0.001 | 1.518 | (1.482 to 1.554) | <0.001 |
| Treatment Category | Operative- GI- other (reference) | 1 |  |  | 1 |  |  |
|  | Operative- laparotomy | 2.869 | (2.627 to 3.134) | <0.001 | 1.15 | (1.095 to 1.208) | <0.001 |
|  | Operative- laparoscopy | 0.274 | (0.181 to 0.414) | <0.001 | 0.305 | (0.279 to 0.332) | <0.001 |
|  | Operative- skin/soft tissue | 1.091 | (1.008 to 1.18) | 0.03 | 0.774 | (0.752 to 0.798) | <0.001 |
|  | Operative- other non-GI | 1.676 | (1.598 to 1.757) | <0.001 | 0.961 | (0.943 to 0.98) | <0.001 |
| Time | Admission Year | 0.918 | (0.914 to 0.922) | <0.001 | 0.964 | (0.962 to 0.965) | <0.001 |
| OR= odds ratio; CI= confidence interval; SURC= Scottish Urban/Rural Classification; CCI= Charlson Comorbidity Index; SIMD= Scottish Index of Multiple Deprivation (1=most deprived; 5=least deprived); GI= gastrointestinal. Model Summary: Cox & Snell R Square=0.034; Nagelkerke R Square=0.241. Number of cases/observations entered into the model: 663586 | | | | | | | |

Table S5: Subgroup Analysis: Admissions with laparotomy. Multiple logistic regression: Inpatient and 1-year mortality as dependent variable, with distance from hospital as covariate of interest.

|  |  | Inpatient Mortality as dependent | | | 1-year Mortality as dependent | | |
| --- | --- | --- | --- | --- | --- | --- | --- |
|  |  | OR | 95% CI | p | OR | 95% CI | p |
| Distance from Hospital (km) | 0 - 2.9km (reference) | 1 |  |  | 1 |  |  |
|  | 2.9 - 6.4km | 0.895 | (0.712 to 1.126) | 0.344 | 0.971 | (0.839 to 1.123) | 0.69 |
|  | 6.4 - 15.2km | 0.87 | (0.69 to 1.095) | 0.235 | 1.034 | (0.895 to 1.194) | 0.654 |
|  | >15.2km | 0.955 | (0.766 to 1.189) | 0.678 | 1.089 | (0.948 to 1.251) | 0.227 |
| Age category | 16-30 (reference) | 1 |  |  | 1 |  |  |
|  | 31-45 | 1.057 | (0.549 to 2.035) | 0.867 | 2.261 | (1.504 to 3.398) | <0.001 |
|  | 46-60 | 2.515 | (1.434 to 4.412) | 0.001 | 5.063 | (3.471 to 7.383) | <0.001 |
|  | 61-75 | 6.504 | (3.81 to 11.101) | <0.001 | 11.244 | (7.772 to 16.269) | <0.001 |
|  | >75 | 12.052 | (7.046 to 20.614) | <0.001 | 24.774 | (17.081 to 35.93) | <0.001 |
| Sex | Female (Male is reference) | 0.858 | (0.734 to 1.005) | 0.057 | 0.887 | (0.805 to 0.978) | 0.016 |
| CCI 10 year | 0; no comorbidity (reference) | 1 |  |  | 1 |  |  |
|  | 1-2; mild comorbidity | 2.974 | (2.387 to 3.706) | <0.001 | 4.175 | (3.67 to 4.75) | <0.001 |
|  | 3-4; moderate comorbidity | 3.599 | (2.716 to 4.768) | <0.001 | 7.914 | (6.703 to 9.344) | <0.001 |
|  | >4; severe comorbidity | 6.517 | (5.064 to 8.385) | <0.001 | 40.383 | (34.304 to 47.539) | <0.001 |
| SIMD Quintile | 5 (reference) | 1 |  |  | 1 |  |  |
|  | 4 | 1.209 | (0.89 to 1.642) | 0.226 | 1.002 | (0.839 to 1.198) | 0.979 |
|  | 3 | 1.371 | (1.023 to 1.836) | 0.034 | 1.138 | (0.959 to 1.351) | 0.14 |
|  | 2 | 1.511 | (1.134 to 2.013) | 0.005 | 1.395 | (1.179 to 1.649) | <0.001 |
|  | 1 | 1.943 | (1.46 to 2.586) | <0.001 | 1.435 | (1.21 to 1.703) | <0.001 |
| Origin | (Domicile is reference) | 1 |  |  | 1 |  |  |
|  | Other | 1.411 | (0.865 to 2.301) | 0.168 | 1.383 | (1.021 to 1.873) | 0.036 |
|  | Transfer | 1.575 | (0.924 to 2.682) | 0.095 | 1.208 | (0.819 to 1.781) | 0.34 |
| Diagnosis Category | High risk (Low Risk is reference) | 2.127 | (1.807 to 2.505) | <0.001 | 1.739 | (1.571 to 1.925) | <0.001 |
| Time | Admission Year | 0.906 | (0.892 to 0.92) | <0.001 | 0.945 | (0.936 to 0.953) | <0.001 |
| OR= odds ratio; CI= confidence interval; SURC= Scottish Urban/Rural Classification; CCI= Charlson Comorbidity Index; SIMD= Scottish Index of Multiple Deprivation (1=most deprived; 5-least deprived); GI= gastrointestinal. Model Summary: Cox & Snell R Square=0.058; Nagelkerke R Square=0.224. Number of cases/observations entered into the model: 20669 | | | | | | | |

Table S6: Subgroup Analysis: Admissions with laparotomy. Multiple logistic regression: Inpatient and 1-year mortality as dependent variable, with 6-Fold SURC as covariate of interest.

|  |  | Inpatient Mortality as dependent | | | 1-year Mortality as dependent | | |
| --- | --- | --- | --- | --- | --- | --- | --- |
|  |  | OR | 95% CI | p | OR | 95% CI | p |
| 6-fold SURC | 1 (reference) | 1 |  |  | 1 |  |  |
|  | 2 | 1.047 | (0.859 to 1.277) | 0.649 | 1.218 | (1.074 to 1.38) | 0.002 |
|  | 3 | 1.019 | (0.747 to 1.389) | 0.908 | 1.147 | (0.946 to 1.391) | 0.162 |
|  | 4 | 1.127 | (0.794 to 1.598) | 0.504 | 1.063 | (0.843 to 1.339) | 0.607 |
|  | 5 | 0.838 | (0.618 to 1.136) | 0.254 | 1.156 | (0.966 to 1.383) | 0.113 |
|  | 6 | 0.964 | (0.703 to 1.323) | 0.822 | 1.147 | (0.941 to 1.399) | 0.174 |
| Age category | 16-30 (reference) | 1 |  |  | 1 |  |  |
|  | 31-45 | 1.062 | (0.552 to 2.044) | 0.857 | 2.265 | (1.507 to 3.404) | <0.001 |
|  | 46-60 | 2.529 | (1.442 to 4.435) | 0.001 | 5.074 | (3.479 to 7.401) | <0.001 |
|  | 61-75 | 6.529 | (3.824 to 11.145) | <0.001 | 11.268 | (7.788 to 16.303) | <0.001 |
|  | >75 | 12.13 | (7.091 to 20.75) | <0.001 | 24.935 | (17.192 to 36.165) | <0.001 |
| Sex | Female (Male is reference) | 0.856 | (0.731 to 1.002) | 0.053 | 0.886 | (0.804 to 0.977) | 0.015 |
| CCI 10 year | 0; no comorbidity (reference) | 1 |  |  | 1 |  |  |
|  | 1-2; mild comorbidity | 2.974 | (2.386 to 3.705) | <0.001 | 4.172 | (3.667 to 4.747) | <0.001 |
|  | 3-4; moderate comorbidity | 3.579 | (2.701 to 4.743) | <0.001 | 7.874 | (6.669 to 9.297) | <0.001 |
|  | >4; severe comorbidity | 6.518 | (5.064 to 8.39) | <0.001 | 40.219 | (34.158 to 47.356) | <0.001 |
| SIMD Quintile | 5 (reference) | 1 |  |  | 1 |  |  |
|  | 4 | 1.255 | (0.917 to 1.719) | 0.156 | 0.998 | (0.83 to 1.199) | 0.983 |
|  | 3 | 1.404 | (1.041 to 1.894) | 0.026 | 1.125 | (0.943 to 1.343) | 0.189 |
|  | 2 | 1.495 | (1.12 to 1.996) | 0.006 | 1.36 | (1.148 to 1.611) | <0.001 |
|  | 1 | 1.916 | (1.439 to 2.551) | <0.001 | 1.425 | (1.201 to 1.691) | <0.001 |
| Origin | (Domicile is reference) | 1 |  |  | 1 |  |  |
|  | Other | 1.413 | (0.866 to 2.305) | 0.166 | 1.397 | (1.031 to 1.893) | 0.031 |
|  | Transfer | 1.579 | (0.928 to 2.687) | 0.092 | 1.235 | (0.839 to 1.819) | 0.284 |
| Diagnosis Category | High risk (Low Risk is reference) | 2.128 | (1.806 to 2.507) | <0.001 | 1.724 | (1.557 to 1.909) | <0.001 |
| Time | Admission Year | 0.906 | (0.892 to 0.921) | <0.001 | 0.944 | (0.935 to 0.952) | <0.001 |
| OR= odds ratio; CI= confidence interval; SURC= Scottish Urban/Rural Classification; CCI= Charlson Comorbidity Index; SIMD= Scottish Index of Multiple Deprivation (1=most deprived, 5=least deprived); GI= gastrointestinal. Model Summary: Cox & Snell R Square=0.058; Nagelkerke R Square=0.224. Number of cases/observations entered into the model: 20669 | | | | | | | |

Table S7. Subgroup Analysis: Transfer patients. Multiple logistic regression: Inpatient and 1-year mortality as dependent variable, with distance to hospital as covariate of interest.

|  |  | Inpatient Mortality as dependent | | | 1-year Mortality as dependent | | |
| --- | --- | --- | --- | --- | --- | --- | --- |
|  |  | OR | 95% CI | p | OR | 95% CI | p |
| Distance from Hospital (km) | 0 - 2.9km (reference) | 1 |  |  | 1 |  |  |
|  | 2.9 - 6.4km | 1.222 | (0.787 to 1.897) | 0.372 | 1.061 | (0.857 to 1.314) | 0.586 |
|  | 6.4 - 15.2km | 1.52 | (1.019 to 2.266) | 0.04 | 1.283 | (1.053 to 1.563) | 0.013 |
|  | >15.2km | 1.239 | (0.865 to 1.773) | 0.242 | 1.259 | (1.059 to 1.497) | 0.009 |
| Age category | 16-30 (reference) | 1 |  |  | 1 |  |  |
|  | 31-45 | 6.719 | (0.864 to 52.245) | 0.069 | 1.993 | (1.333 to 2.981) | 0.001 |
|  | 46-60 | 15.535 | (2.129 to 113.347) | 0.007 | 4.297 | (2.967 to 6.224) | <0.001 |
|  | 61-75 | 31.153 | (4.321 to 224.582) | 0.001 | 8.186 | (5.69 to 11.777) | <0.001 |
|  | >75 | 59.925 | (8.327 to 431.232) | <0.001 | 17.358 | (12.044 to 25.015) | <0.001 |
| Sex | Female (Male is reference) | 1.196 | (0.968 to 1.477) | 0.097 | 0.96 | (0.866 to 1.065) | 0.444 |
| CCI 10 year | 0; no comorbidity (reference) | 1 |  |  | 1 |  |  |
|  | 1-2; mild comorbidity | 2.098 | (1.449 to 3.036) | <0.001 | 3.657 | (3.108 to 4.303) | <0.001 |
|  | 3-4; moderate comorbidity | 3.208 | (2.181 to 4.72) | <0.001 | 6.538 | (5.483 to 7.796) | <0.001 |
|  | >4; severe comorbidity | 5.293 | (3.648 to 7.679) | <0.001 | 17.39 | (14.603 to 20.708) | <0.001 |
| SIMD Quintile | 5 (reference) | 1 |  |  | 1 |  |  |
|  | 4 | 1.264 | (0.822 to 1.946) | 0.286 | 1.005 | (0.815 to 1.24) | 0.961 |
|  | 3 | 1.128 | (0.75 to 1.697) | 0.563 | 1.104 | (0.907 to 1.343) | 0.323 |
|  | 2 | 0.912 | (0.599 to 1.387) | 0.667 | 1.059 | (0.871 to 1.287) | 0.567 |
|  | 1 | 1.159 | (0.759 to 1.77) | 0.496 | 1.189 | (0.973 to 1.453) | 0.091 |
| Diagnosis Category | High risk (Low Risk is reference) | 2.706 | (2.118 to 3.457) | <0.001 | 1.646 | (1.432 to 1.892) | <0.001 |
| Treatment Category | Non-operative (reference) | 1 |  |  | 1 |  |  |
|  | Operative- laparotomy | 1.164 | (0.669 to 2.027) | 0.591 | 0.727 | (0.497 to 1.065) | 0.101 |
|  | Operative- laparoscopy | 0 | (0 to .) | 0.995 | 0.529 | (0.265 to 1.056) | 0.071 |
|  | Operative- GI- other | 0.309 | (0.227 to 0.421) | <0.001 | 0.938 | (0.826 to 1.065) | 0.325 |
|  | Operative- skin/soft tissue | 0.269 | (0.14 to 0.518) | <0.001 | 0.761 | (0.602 to 0.961) | 0.022 |
|  | Operative- other non-GI | 0.875 | (0.667 to 1.148) | 0.335 | 0.833 | (0.717 to 0.967) | 0.017 |
| Time | Admission Year | 0.907 | (0.891 to 0.923) | <0.001 | 0.93 | (0.923 to 0.938) | <0.001 |
| OR= odds ratio; CI= confidence interval; SURC= Scottish Urban/Rural Classification; CCI= Charlson Comorbidity Index; SIMD= Scottish Index of Multiple Deprivation (1=most deprived; 5=least deprived); GI= gastrointestinal. Model Summary: Cox & Snell R Square=0.058; Nagelkerke R Square=0.228. Number of cases/observations entered into the model: 11869 | | | | | | | |

Table S8. Subgroup Analysis: Transfer patients. Multiple logistic regression: Inpatient and 1-year mortality as dependent variable, with 6-Fold SURC as covariate of interest.

|  |  | Inpatient Mortality as dependent | | | 1-year Mortality as dependent | | |
| --- | --- | --- | --- | --- | --- | --- | --- |
|  |  | OR | 95% CI | p | OR | 95% CI | p |
| 6-fold SURC | 1 (reference) | 1 |  |  | 1 |  |  |
|  | 2 | 0.884 | (0.637 to 1.226) | 0.459 | 1.017 | (0.869 to 1.191) | 0.831 |
|  | 3 | 1.254 | (0.8 to 1.966) | 0.324 | 1.272 | (1.016 to 1.592) | 0.036 |
|  | 4 | 1.463 | (0.951 to 2.248) | 0.083 | 1.287 | (1.02 to 1.624) | 0.033 |
|  | 5 | 1.011 | (0.646 to 1.582) | 0.962 | 1.25 | (1.01 to 1.546) | 0.04 |
|  | 6 | 1.291 | (0.874 to 1.907) | 0.2 | 1.034 | (0.844 to 1.267) | 0.748 |
| Age category | 16-30 (reference) | 1 |  |  | 1 |  |  |
|  | 31-45 | 6.597 | (0.848 to 51.295) | 0.071 | 1.975 | (1.321 to 2.952) | 0.001 |
|  | 46-60 | 15.316 | (2.099 to 111.761) | 0.007 | 4.24 | (2.929 to 6.139) | <0.001 |
|  | 61-75 | 30.051 | (4.168 to 216.664) | 0.001 | 7.986 | (5.554 to 11.482) | <0.001 |
|  | >75 | 55.866 | (7.763 to 402.064) | <0.001 | 16.586 | (11.518 to 23.885) | <0.001 |
| Sex | Female (Male is reference) | 1.184 | (0.958 to 1.463) | 0.118 | 0.961 | (0.866 to 1.065) | 0.446 |
| CCI 10 year | 0; no comorbidity (reference) | 1 |  |  | 1 |  |  |
|  | 1-2; mild comorbidity | 2.078 | (1.435 to 3.008) | <0.001 | 3.673 | (3.122 to 4.322) | <0.001 |
|  | 3-4; moderate comorbidity | 3.191 | (2.169 to 4.694) | <0.001 | 6.641 | (5.571 to 7.916) | <0.001 |
|  | >4; severe comorbidity | 5.265 | (3.628 to 7.639) | <0.001 | 17.798 | (14.956 to 21.18) | <0.001 |
| SIMD Quintile | 5 (reference) | 1 |  |  | 1 |  |  |
|  | 4 | 1.159 | (0.742 to 1.811) | 0.517 | 0.984 | (0.793 to 1.222) | 0.887 |
|  | 3 | 1.013 | (0.661 to 1.553) | 0.954 | 1.103 | (0.899 to 1.352) | 0.347 |
|  | 2 | 0.883 | (0.576 to 1.354) | 0.569 | 1.047 | (0.858 to 1.277) | 0.652 |
|  | 1 | 1.199 | (0.784 to 1.836) | 0.402 | 1.197 | (0.978 to 1.464) | 0.081 |
| Diagnosis Category | High risk (Low Risk is reference) | 2.662 | (2.084 to 3.401) | <0.001 | 1.66 | (1.444 to 1.908) | <0.001 |
| Treatment Category | Non-operative (reference) | 1 |  |  | 1 |  |  |
|  | Operative- laparotomy | 1.12 | (0.642 to 1.952) | 0.69 | 0.755 | (0.516 to 1.105) | 0.148 |
|  | Operative- laparoscopy | 0 | (0 to .) | 0.995 | 0.519 | (0.26 to 1.037) | 0.063 |
|  | Operative- GI- other | 0.305 | (0.224 to 0.414) | <0.001 | 0.956 | (0.843 to 1.084) | 0.481 |
|  | Operative- skin/soft tissue | 0.259 | (0.134 to 0.498) | <0.001 | 0.768 | (0.608 to 0.969) | 0.026 |
|  | Operative- other non-GI | 0.813 | (0.619 to 1.069) | 0.138 | 0.842 | (0.724 to 0.979) | 0.025 |
| Time | Admission Year | 0.907 | (0.891 to 0.924) | <0.001 | 0.929 | (0.921 to 0.936) | <0.001 |
| OR= odds ratio; CI= confidence interval; SURC= Scottish Urban/Rural Classification; CCI= Charlson Comorbidity Index; SIMD= Scottish Index of Multiple Deprivation (1=most deprived; 5=least deprived); GI= gastrointestinal. Model Summary: Cox & Snell R Square=0.059; Nagelkerke R Square=0.230. Number of cases/observations entered into the model: 11869 | | | | | | | |

Table S9. STROBE Statement—Checklist of items that should be included in reports of cohort studies.^25^

|  | Item No | Recommendation | Location (Page) |
| --- | --- | --- | --- |
| **Title and abstract** | 1 | (*a*) Indicate the study’s design with a commonly used term in the title or the abstract | 1 |
|  |  | (*b*) Provide in the abstract an informative and balanced summary of what was done and what was found | 2 |
| Introduction | | |  |
| Background/rationale | 2 | Explain the scientific background and rationale for the investigation being reported | 3 |
| Objectives | 3 | State specific objectives, including any prespecified hypotheses | 3 |
| Methods | | |  |
| Study design | 4 | Present key elements of study design early in the paper | 4 |
| Setting | 5 | Describe the setting, locations, and relevant dates, including periods of recruitment, exposure, follow-up, and data collection | 4 |
| Participants | 6 | (*a*) Give the eligibility criteria, and the sources and methods of selection of participants. Describe methods of follow-up | 4 |
|  |  | (*b*) For matched studies, give matching criteria and number of exposed and unexposed | NA |
| Variables | 7 | Clearly define all outcomes, exposures, predictors, potential confounders, and effect modifiers. Give diagnostic criteria, if applicable | 4,5 |
| Data sources/ measurement | 8* | For each variable of interest, give sources of data and details of methods of assessment (measurement). Describe comparability of assessment methods if there is more than one group | 4,5 |
| Bias | 9 | Describe any efforts to address potential sources of bias | 5 |
| Study size | 10 | Explain how the study size was arrived at | 4,6 |
| Quantitative variables | 11 | Explain how quantitative variables were handled in the analyses. If applicable, describe which groupings were chosen and why | 4,5 |
| Statistical methods | 12 | (*a*) Describe all statistical methods, including those used to control for confounding | 5 |
|  |  | (*b*) Describe any methods used to examine subgroups and interactions | 5 |
|  |  | (*c*) Explain how missing data were addressed | 6 |
|  |  | (*d*) If applicable, explain how loss to follow-up was addressed | NA |
|  |  | (*e*) Describe any sensitivity analyses | 5 |
| Results | | |  |
| Participants | 13* | (a) Report numbers of individuals at each stage of study—eg numbers potentially eligible, examined for eligibility, confirmed eligible, included in the study, completing follow-up, and analysed | 6 |
|  |  | (b) Give reasons for non-participation at each stage | 6 |
|  |  | (c) Consider use of a flow diagram | NA |
| Descriptive data | 14* | (a) Give characteristics of study participants (eg demographic, clinical, social) and information on exposures and potential confounders | 6 |
|  |  | (b) Indicate number of participants with missing data for each variable of interest | 6 |
|  |  | (c) Summarise follow-up time (eg, average and total amount) | 4 |
| Outcome data | 15* | Report numbers of outcome events or summary measures over time | 6, 15, 16 |
| Main results | 16 | (*a*) Give unadjusted estimates and, if applicable, confounder-adjusted estimates and their precision (eg, 95% confidence interval). Make clear which confounders were adjusted for and why they were included | 17-20 |
|  |  | (*b*) Report category boundaries when continuous variables were categorized | 14-20 |
|  |  | (*c*) If relevant, consider translating estimates of relative risk into absolute risk for a meaningful time period | NA |
| Other analyses | 17 | Report other analyses done—eg analyses of subgroups and interactions, and sensitivity analyses | 6,7 |
| Discussion | | |  |
| Key results | 18 | Summarise key results with reference to study objectives | 7,8 |
| Limitations | 19 | Discuss limitations of the study, taking into account sources of potential bias or imprecision. Discuss both direction and magnitude of any potential bias | 8,9 |
| Interpretation | 20 | Give a cautious overall interpretation of results considering objectives, limitations, multiplicity of analyses, results from similar studies, and other relevant evidence | 8,9 |
| Generalisability | 21 | Discuss the generalisability (external validity) of the study results | 8-10 |
| Other information | | |  |
| Funding | 22 | Give the source of funding and the role of the funders for the present study and, if applicable, for the original study on which the present article is based | 1 |

*Give information separately for exposed and unexposed groups.

**Note:** An Explanation and Elaboration article discusses each checklist item and gives methodological background and published examples of transparent reporting. The STROBE checklist is best used in conjunction with this article (freely available on the Web sites of PLoS Medicine at http://www.plosmedicine.org/, Annals of Internal Medicine at http://www.annals.org/, and Epidemiology at http://www.epidem.com/). Information on the STROBE Initiative is available at http://www.strobe-statement.org.

Table S10. Inpatient Mortality by Distance to Hospital (quartiles), for Scottish EGS admissions 1998-2018.

| Distance to Hospital |  | 0-2.9km |  | 2.9-6.4km |  | 6.4-15.2km |  | >15.2km |  |
| --- | --- | --- | --- | --- | --- | --- | --- | --- | --- |
|  |  | Died / alive | % | Died / alive | % | Died / alive | % | Died / alive | % |
| Age Category | 16-30 | 25 / 78722 | 0.0% | 23 / 75648 | 0.0% | 20 / 71171 | 0.0% | 23 / 66355 | 0.0% |
|  | 31-45 | 126 / 84562 | 0.1% | 111 / 85666 | 0.1% | 116 / 81495 | 0.1% | 84 / 73865 | 0.1% |
|  | 46-60 | 571 / 80797 | 0.7% | 580 / 85288 | 0.7% | 581 / 87008 | 0.7% | 459 / 85139 | 0.5% |
|  | 61-75 | 2065 / 77885 | 2.7% | 2045 / 79508 | 2.6% | 1917 / 84552 | 2.3% | 1737 / 90749 | 1.9% |
|  | >75 | 3878 / 64564 | 6.0% | 3691 / 60361 | 6.1% | 3678 / 62532 | 5.9% | 3750 / 70849 | 5.3% |
| Sex | Male | 3015 / 185729 | 1.6% | 2899 / 180568 | 1.6% | 2879 / 178361 | 1.6% | 2829 / 186802 | 1.5% |
|  | Female | 3650 / 200801 | 1.8% | 3551 / 205903 | 1.7% | 3433 / 208397 | 1.6% | 3224 / 200155 | 1.6% |
| CCI 10 year | 0; no comorbidity | 624 / 226452 | 0.3% | 571 / 226750 | 0.3% | 566 / 227314 | 0.2% | 622 / 213750 | 0.3% |
|  | 1-2; mild comorbidity | 2167 / 104790 | 2.1% | 2069 / 103236 | 2.0% | 2062 / 102244 | 2.0% | 2035 / 106640 | 1.9% |
|  | 3-4; moderate comorbidity | 1327 / 29821 | 4.4% | 1359 / 30006 | 4.5% | 1298 / 30019 | 4.3% | 1299 / 33673 | 3.9% |
|  | >4; severe comorbidity | 2547 / 25467 | 10.0% | 2451 / 26479 | 9.3% | 2386 / 27181 | 8.8% | 2097 / 32894 | 6.4% |
| SIMD Quintile | 1 | 2321 / 141108 | 1.6% | 2393 / 149746 | 1.6% | 1626 / 105357 | 1.5% | 778 / 55898 | 1.4% |
|  | 2 | 1622 / 95070 | 1.7% | 1572 / 87040 | 1.8% | 1552 / 89171 | 1.7% | 1434 / 85629 | 1.7% |
|  | 3 | 1174 / 60619 | 1.9% | 878 / 56551 | 1.6% | 1282 / 71402 | 1.8% | 1794 / 112433 | 1.6% |
|  | 4 | 782 / 45118 | 1.7% | 835 / 44118 | 1.9% | 1057 / 67633 | 1.6% | 1420 / 89870 | 1.6% |
|  | 5 | 766 / 44615 | 1.7% | 772 / 49016 | 1.6% | 795 / 53195 | 1.5% | 627 / 43127 | 1.5% |
| Origin | Domicile | 6178 / 371283 | 1.7% | 6069 / 374794 | 1.6% | 5862 / 375253 | 1.6% | 5226 / 359706 | 1.5% |
|  | Other | 283 / 11745 | 2.4% | 216 / 8602 | 2.5% | 241 / 7914 | 3.0% | 421 / 18967 | 2.2% |
|  | Transfer | 42 / 1540 | 2.7% | 49 / 1764 | 2.8% | 81 / 2332 | 3.5% | 229 / 5832 | 3.9% |
| High Risk Diagnosis | No | 4980 / 363115 | 1.4% | 4634 / 360942 | 1.3% | 4660 / 359617 | 1.3% | 4249 / 354892 | 1.2% |
|  | Yes | 1685 / 23415 | 7.2% | 1816 / 25529 | 7.1% | 1652 / 27141 | 6.1% | 1804 / 32065 | 5.6% |
| Treatment Categories | Non-operative | 4051 / 223014 | 1.8% | 4095 / 228419 | 1.8% | 3952 / 232097 | 1.7% | 3403 / 206160 | 1.7% |
|  | Operative- GI- other | 808 / 61136 | 1.3% | 806 / 63104 | 1.3% | 880 / 64502 | 1.4% | 793 / 70658 | 1.1% |
|  | Operative- other non-GI | 1403 / 66637 | 2.1% | 1168 / 57638 | 2.0% | 1119 / 53140 | 2.1% | 1397 / 69730 | 2.0% |
|  | Operative- skin/soft tissue | ** / 23728 |  | 213 / 22908 | 0.9% | 197 / 21661 | 0.9% | ** / 23158 |  |
|  | Operative- laparoscopy | * / 7477 |  | 11 / 9492 | 0.1% | 6 / 10549 | 0.1% | * / 11476 |  |
|  | Operative- laparotomy | 182 / 4538 | 4.0% | 157 / 4910 | 3.2% | 158 / 4809 | 3.3% | 235 / 5775 | 4.1% |
| 6-Fold SURC | 1 | 3585 / 205989 | 1.7% | 3530 / 219021 | 1.6% | 1377 / 99025 | 1.4% | 69 / 10230 | 0.7% |
|  | 2 | 2575 / 148251 | 1.7% | 2348 / 133615 | 1.8% | 2800 / 165871 | 1.7% | 2111 / 133967 | 1.6% |
|  | 3 | 20 / 1357 | 1.5% | 196 / 12927 | 1.5% | 976 / 55064 | 1.8% | 830 / 49909 | 1.7% |
|  | 4 | 357 / 24543 | 1.5% | * / 220 |  | ** / 2517 |  | 695 / 41439 | 1.7% |
|  | 5 | 89 / 4840 | 1.8% | 344 / 18118 | 1.9% | 837 / 51629 | 1.6% | 942 / 67198 | 1.4% |
|  | 6 | 39 / 1550 | 2.5% | ** / 2570 |  | ** / 12652 |  | 1406 / 84214 | 1.7% |
| *number is less than or equal to 5. **number is greater than 5 but removed so that a small number in the same row cannot be identified. CCI= Charlson Comorbidity Index; SIMD= Scottish Index of Multiple Deprivation (1=most deprived; 5=least deprived), ICD-10= International Statistical Classification of Diseases and Related Health Problems 10th Revision; OPCS4= Office of Population Censuses and Surveys Classification of Interventions and Procedures 4th Revision; GI= gastrointestinal; SURC= Scottish Urban/Rural Classification. | | | | | | | | | |

Table S11. Inpatient Mortality by 6-Fold Scottish Urban/Rural Classification (SURC), for Scottish EGS admissions 1998-2018.

|  | |  |  |  |  |  |  |
| --- | --- | --- | --- | --- | --- | --- | --- |
| 6-Fold SURC |  | 1 | 2 | 3 | 4 | 5 | 6 |
|  |  | Died / alive (%) | Died / alive (%) | Died / alive (%) | Died / alive (%) | Died / alive (%) | Died / alive (%) |
| Age Category | 16-30 | 32 / 110739 (0.0) | 38 / 110705 (0.0) | 8 / 21279 (0.0) | * / 11932 | * / 22940 | * / 14301 |
|  | 31-45 | 131 / 119583 (0.1) | 192 / 125938 (0.2) | 41 / 23906 (0.2) | ** / 12342 | ** / 27534 | ** / 16285 |
|  | 46-60 | 752 / 116013 (0.6) | 869 / 127557 (0.7) | 176 / 25985 (0.7) | 76 / 13754 (0.6) | 188 / 33205 (0.6) | 130 / 21718 (0.6) |
|  | 61-75 | 2643 / 105492 (2.5) | 3111 / 124658 (2.5) | 600 / 27136 (2.2) | 296 / 15709 (1.9) | 648 / 33793 (1.9) | 466 / 25906 (1.8) |
|  | >75 | 5003 / 82438 (6.1) | 5624 / 92846 (6.1) | 1197 / 20951 (5.7) | 730 / 14982 (4.9) | 1341 / 24313 (5.5) | 1102 / 22776 (4.8) |
| Sex | Male | 3822 / 250711 (1.5) | 4456 / 272430 (1.6) | 923 / 55491 (1.7) | 517 / 33649 (1.5) | 1054 / 68664 (1.5) | 850 / 50515 (1.7) |
|  | Female | 4739 / 283554 (1.7) | 5378 / 309274 (1.7) | 1099 / 63766 (1.7) | 606 / 35070 (1.7) | 1158 / 73121 (1.6) | 878 / 50471 (1.7) |
| CCI 10 year | CCI=0; no comorbidity | 802 / 314962 (0.3) | 855 / 334791 (0.3) | 194 / 68281 (0.3) | 122 / 37780 (0.3) | 238 / 82907 (0.3) | 172 / 55545 (0.3) |
|  | CCI=1,2; mild comorbidity | 2811 / 141483 (2.0) | 3207 / 158027 (2.0) | 624 / 32207 (1.9) | 399 / 19534 (2.0) | 728 / 37359 (1.9) | 564 / 28300 (2.0) |
|  | CCI=3, 4; moderate comorbidity | 1808 / 41571 (4.3) | 1987 / 46562 (4.3) | 425 / 9674 (4.4) | 235 / 6147 (3.8) | 448 / 10698 (4.2) | 380 / 8867 (4.3) |
|  | CCI>4; severe comorbidity | 3140 / 36249 (8.7) | 3785 / 42324 (8.9) | 779 / 9095 (8.6) | 367 / 5258 (7.0) | 798 / 10821 (7.4) | 612 / 8274 (7.4) |
| SIMD Quintile | 1 | 3611 / 229757 (1.6) | 2857 / 181333 (1.6) | 318 / 20024 (1.6) | 128 / 8926 (1.4) | 152 / 8760 (1.7) | 52 / 3309 (1.6) |
|  | 2 | 1778 / 102401 (1.7) | 2908 / 168363 (1.7) | 491 / 26457 (1.9) | 334 / 20965 (1.6) | 381 / 23257 (1.6) | 288 / 15467 (1.9) |
|  | 3 | 968 / 62578 (1.5) | 1833 / 99893 (1.8) | 534 / 29343 (1.8) | 326 / 19805 (1.6) | 680 / 42529 (1.6) | 787 / 46857 (1.7) |
|  | 4 | 902 / 56015 (1.6) | 1192 / 66064 (1.8) | 341 / 21623 (1.6) | 271 / 15232 (1.8) | 828 / 54765 (1.5) | 560 / 33040 (1.7) |
|  | 5 | 1302 / 83514 (1.6) | 1044 / 66051 (1.6) | 338 / 21810 (1.5) | 64 / 3791 (1.7) | 171 / 12474 (1.4) | 41 / 2313 (1.8) |
| Origin | Domicile | 8104 / 519247 (1.6) | 9027 / 556497 (1.6) | 1827 / 114261 (1.6) | 938 / 63158 (1.5) | 1990 / 135009 (1.5) | 1449 / 92864 (1.6) |
|  | Other | 249 / 11840 (2.1) | 468 / 17297 (2.7) | 104 / 3401 (3.1) | 95 / 4226 (2.2) | 115 / 4591 (2.5) | 130 / 5873 (2.2) |
|  | Transfer | 62 / 1621 (3.8) | 132 / 5586 (2.4) | 35 / 931 (3.8) | 46 / 709 (6.5) | 38 / 1193 (3.2) | 88 / 1428 (6.2) |
| High Risk Diagnosis | No | 6170 / 500384 (1.2) | 7206 / 540148 (1.3) | 1447 / 109952 (1.3) | 823 / 64205 (1.3) | 1574 / 130119 (1.2) | 1303 / 93758 (1.4) |
|  | Yes | 2391 / 33881 (7.1) | 2628 / 41556 (6.3) | 575 / 9305 (6.2) | 300 / 4514 (6.6) | 638 / 11666 (5.5) | 425 / 7228 (5.9) |
| Treatment Categories | Non-operative | 5436 / 317603 (1.7) | 6085 / 339011 (1.8) | 1252 / 67391 (1.9) | 566 / 37109 (1.5) | 1281 / 77327 (1.7) | 881 / 51249 (1.7) |
|  | Operative- GI- other | 1075 / 85527 (1.3) | 1324 / 100770 (1.3) | 251 / 21586 (1.2) | 135 / 9948 (1.4) | 279 / 25675 (1.1) | 223 / 15894 (1.4) |
|  | Operative- other non-GI | 1571 / 79015 (2.0) | 1826 / 85863 (2.1) | 393 / 18293 (2.1) | 333 / 15723 (2.1) | 482 / 23643 (2.0) | 482 / 24608 (2.0) |
|  | Operative- skin/soft tissue | 262 / 32230 (0.8) | 322 / 34304 (0.9) | ** / 7016 | ** / 3675 | ** / 8402 | ** / 5828 |
|  | Operative- laparoscopy | 7 / 13234 (0.1) | 9 / 14810 (0.1) | * / 3335 | * / 1314 | * / 4411 | * / 1890 |
|  | Operative- laparotomy | 210 / 6656 (3.2) | 268 / 6946 (3.9) | 62 / 1636 (3.8) | 48 / 950 (5.1) | 71 / 2327 (3.1) | 73 / 1517 (4.8) |
| *number is less than or equal to 5. **number is greater than 5 but removed so that a small number in the same row cannot be identified. CCI= Charlson Comorbidity Index; SIMD= Scottish Index of Multiple Deprivation (1=most deprived; 5=least deprived), ICD-10= International Statistical Classification of Diseases and Related Health Problems 10th Revision; OPCS4= Office of Population Censuses and Surveys Classification of Interventions and Procedures 4th Revision; GI= gastrointestinal; SURC= Scottish Urban/Rural Classification. | | | | | | | |
